# Intention to Vaccinate against COVID-19 among Young Adults: The Role of Conspiratorial Thinking

**DOI:** 10.3390/vaccines11020321

**Published:** 2023-01-31

**Authors:** Ivana Hromatko, Una Mikac, Meri Tadinac

**Affiliations:** Department of Psychology, Faculty of Humanities and Social Sciences, University of Zagreb, 10000 Zagreb, Croatia

**Keywords:** vaccine hesitancy, COVID-19 vaccine, conspiratorial thinking, young adults

## Abstract

The anti-scientific and anti-vaccine movements gained momentum amidst the health and socio-economic crisis brought about by the COVID-19 pandemic. These widespread pseudoscientific beliefs and the endorsement of conspiracy theories likely contributed to the COVID-19 vaccine hesitancy. The aim of this study was to explore which variables best differentiated between groups of vaccinated (n = 289), vaccine-hesitant (n = 106), and vaccine-refusing (n = 146) young adults. The study was conducted online at the beginning of the mass vaccination campaign in Croatia when the vaccine just became available for younger and non-vulnerable members of the general population. The demographic variables, COVID-19 anxiety, and conspiratorial thinking regarding COVID-19 were entered into the discriminant analysis. The function explaining 89.2% of the group differences, mostly between the vaccinated and vaccine-refusing, was largely defined by conspiratorial thinking regarding COVID-19 (0.852), followed by variables with substantially less discriminative power, including COVID-19 anxiety (0.423; lower in the vaccine-refusing group), political orientation (0.486; vaccine-refusing leaning less to the left), financial and educational status (0.435 and 0.304, respectively; both lower in the vaccine-refusing group), and religiosity (0.301; higher in the vaccine-refusing group). These results confirm that among young adults, the decision to vaccinate against COVID-19 might be heavily influenced by one’s proclivity to engage in conspiratorial thinking.

## 1. Introduction

Since the outbreak of the COVID-19 pandemic, health authorities worldwide have been dealing with yet another sort of a pandemic, paralleling the viral one both in contagiousness and its potential to inflict harmful consequences: the COVID-19 infodemic [1]. Aggravated by the pandemic and mistrust in governments, scientific institutions, health authorities, and the media, public opinion is strongly ridden regarding who and what are the reliable sources [2,3,4,5,6,7,8,9]. Large-scale surveys show that 20–30% of respondents believe that the government institutions and the mainstream media actively participate in hiding the truth pertaining to the COVID-19 pandemic, for example, that some powerful people intentionally planned the COVID-19 outbreak or that the pandemic was a part of a scheme by global elites to profit off of infectious diseases, e.g., [10,11]. Some respondents believe that COVID-19 is part of a government bioweapons program, that 5G cell towers are spreading COVID-19, or that pharmaceutical companies are encouraging the spread of COVID-19 for profit-making purposes (building upon a well-known fallacy, the argumentum ad big pharma; [12]). Interestingly, Freeman et al. [10] found a high correlation between the specific and generic coronavirus conspiracy component, implying that there is a shared factorial space for generic and specific conspiratorial cognitions regarding COVID-19.

The appeal of conspiracy theories is not a new phenomenon, especially not at times of crises, and the consequences of the COVID-19 pandemic certainly amount to making it a global crisis-level event. The endorsement of conspiracy theories occurs when official narratives are experienced as deficient and obscure, while events are viewed as deceitful, with non-conclusive explanations [13]. Some authors argue that endorsing such explanations provides an illusion of control and has a soothing effect of reassuring oneself that disasters of such magnitude do not just happen at random, but have a tangible cause [14,15]. It has been shown that conspiratorial mentality was linked to the refusal to trust science, to the disregard of the biomedical model of disease, and legal means of political engagement [16]. Unfortunately, epistemically suspect beliefs, such as pseudoscientific beliefs or the endorsement of conspiracy theories, can have far-reaching negative real-life outcomes. Even prior to these COVID-19 pandemic trends, public consent to health authorities’ guidelines during viral diseases outbreaks has been difficult to achieve [17,18], and the rise in global anti-vaccination and anti-scientific movements [19,20] likely contributed to the widespread lack of adherence to both non-pharmacological (mask wearing, social distancing, regular hand washing, etc.) and pharmacological (vaccination) preventative epidemiologic recommendations [16,21,22,23] during the COVID-19 pandemic. In this particular context, conspiratorial thinking and various cognitive fallacies (see, e.g., Azarpanah et al. [24], who identified 15 possible cognitive fallacies contributing to the vaccine hesitancy) easily add momentum to the vaccine hesitancy movement. Thus, even though vaccine development is more transparent than ever, conspiracy theories continue to thrive [8,25].

At the same time, health anxiety and related mental issues have skyrocketed since the beginning of the COVID-19 pandemic [26]. This increase in anxiety has been attributed to various factors, such as official measures and (dis)trust in official sources of information, personal experiences with COVID-19, and the perception of the threat of COVID-19 (see, e.g., [27]). From a rational and self-preservation point of view, vaccination would seem to be a logical choice, at least for those individuals whose mental health deterioration is induced by COVID-19-related health anxiety. Indeed, it has been shown that vaccination against COVID-19 alleviates disease-related stress and anxiety, especially among the most vulnerable groups [28,29]. Yet, acceptance rates are often far from satisfactory [30,31,32,33,34,35,36,37,38,39]. A cross-cultural comparison of COVID-19 vaccine hesitancy conducted in June 2021 [40] showed an overall hesitancy rate of 24.8%, but with substantial variations ranging from 2.5 in China to 48.4% in Russia. In the majority of countries where the study was conducted, the hesitancy rate ranged between 20 and 40%, including in the US, Canada, and most European countries.

The comparison across multiple studies is somewhat complicated by the fact that various researchers operationalize the term “vaccine hesitancy” differently [41]. It has also been noted that there are multiple phases of vaccine hesitancy (vaccine eagerness, vaccine ignorance, vaccine resistance, vaccine confidence, vaccine complacency, and vaccine apathy), which may be sequential, but can also co-exist at the same time in different regions and at different times in the same region [42]. Generally, mistrust in vaccines spreads across several domains: mistrust in benefits, worries of unforeseen effects, preference for natural immunity, and concerns about profiteering [43]. Claims stemming from all of these domains have been heard abundantly across various media outlets and social networks since the vaccines against SARS-CoV-2 became widely available. In lieu of such sentiments, misperceptions of vaccine safety, efficacy, and risks, as well as mistrust in health authorities and institutions in charge of vaccination campaigns, have been contributing to vaccine hesitancy [40,44]. However, in the light of the fact that vaccination remains one of the most effective interventions to control the pandemic, the reasons for the widespread refusal of the available vaccines remain rather elusive. 

In this particular study, we opted not to measure attitudes toward COVID-19 vaccine but rather categorized participants according to their vaccination status and their intent to be vaccinated in the future. Previous studies have already addressed the predictors of COVID-19 vaccine acceptance, and a recent review and meta-analysis [45] showed that the factors associated with a higher COVID-19 vaccine acceptance included a greater perceived risk of COVID-19, a lower level of perceived vaccine harm, higher educational attainment and household income, an older age, and being of a White ethnicity and male sex. The aim of this study was to further deepen the understanding of specific factors by analyzing which of them contribute most to the differentiation between groups of vaccine-hesitant, vaccine-refusing, and vaccinated young adults. Along with socio-demographic characteristics, we included measures of COVID-19-related anxiety and conspiratorial thinking regarding COVID-19, as these two factors presumably have the opposite effects on the decision to be vaccinated. 

## 2. Materials and Methods

### 2.1. Data Collection and Participants

The epidemic in Croatia was officially declared in March of 2020, followed by a lockdown, including all educational and cultural institutions and events and restrictions on public gatherings and travels, lasting for two months [46]. The second wave, with more than 4000 hospitalizations and almost 1000 casualties, occurred between August of 2020 and February of 2021, at the end of which vaccination began, at first aimed at vulnerable and at-risk groups. At the time of the data collection, the third wave, with more than 3000 hospitalizations and about 50 casualties, was at its end and about 50% of the adult population was vaccinated [47]. In this period, in June 2021, the implementation of the EUs digital COVID certificate system began, allowing those vaccinated access to various public institutions and events, together with pro-vaccination public campaigns. Delta and omicron variants appeared in the second half of 2021 in the time of the fourth and the fifth wave and after the data collection.

The data were collected during the three months in the summer of 2021 with an online questionnaire on SoSci Survey in Croatia (the questionnaire was a part of a larger research project and included additional measures not presented in this paper, and different parts of the questionnaire were presented to different respondents depending on their previous responses; it took about 15 min to fill out). The invitation to participate in the research was shared on social media pages and applications aimed at the general population and at those looking for dating opportunities, e.g., Facebook, forums, and Badoo. Informed consent was obtained from the participants before the start of the questionnaire. The questionnaire was accessed by 893 individuals, constituting a combination of a convenience and snowball sample, of which 481 responded to the questions relevant for this research. This sample of 481 respondents was then used in the data analysis. The study was approved by the Ethics Committee of the Department of Psychology (Faculty of Humanities and Social Sciences, University of Zagreb, Zagreb, Croatia; code EPOP-2022-23-10).

### 2.2. Measures

#### 2.2.1. Sociodemographic Data

We collected data on age, gender, and relationship and parenthood status at the beginning of the questionnaire and at the end of the questionnaire, we collected data on employment status (*pupils*, *students*, *employed*, *unemployed*, *retired*), attained education level (*primary*, *high school*, *college*, *bachelor*, *master, doctoral degree*), size of place of residence, household income (is it perceived as below/above average or average), and household members. Here, we also collected data on the importance of religion and political orientation on a scale from 1 to 100. Both variables showed trimodal distributions, with the highest frequencies of responses at the extremes and in the center, so both scales were recoded into three levels: low (1–33), mid (34–66), and high (67–100) importance of religion, and left (1–33), center (34–66), and right (67–100) political orientation. Regarding COVID-19, in the central part of the questionnaire, we asked the participants if they had COVID-19 previously (with responses *Yes*/ *No*/*I don’t know*), if they were vaccinated (with responses *Yes, with all prescribed doses*/*Yes, but not with the other dose*/*No*), and if they were not, and whether they intended to become vaccinated (with responses *Yes*/*No*/*I’m unsure*). Based on these items, we discerned participants with different intentions to vaccinate.

#### 2.2.2. COVID-19-Related Anxiety

We used the COVID-19 Anxiety Scale (CAS-5; [48]) in the central part of the questionnaire after asking the questions regarding COVID-19. The scale was composed of five items assessing the participants’ concerns about COVID-19, e.g., perceived severity of infection in case of contracting COVID-19. The participants rated the extent to which each item related to them on a 5-point scale ranging from 1 (*not at all*) to 5 (*very much*) and the total result was expressed as their average. The scale showed good psychometric characteristics [49] and the reliability in our sample was Cronbach α = 0.748/McDonald’s ω = 0.752.

#### 2.2.3. Conspiratorial Thinking Regarding COVID-19

To assess conspiratorial thinking regarding COVID-19, we constructed a scale which consisted of four items from Egorova et al. [50] and two from Tonković et al. [7]. Exploratory factor analysis with principal axis factors indicated the existence of one factor explaining 55.6% of the variance, with loadings between 0.52 and 0.84. The six items were rated on a 5-point scale ranging from 1 (*Don’t agree at all*) to 5 (*Agree completely*) and the total result was expressed as their average. They included items on vaccinations being a way to implant a chip, coronavirus being similar to flu, the pandemic/virus being a lie and a way to distract from state problems, to limit personal freedoms, and to profit. The reliability in our sample was Cronbach α = 0.878/McDonald’s ω = 0.880. The scale was presented after the COVID-19 Anxiety Scale.

### 2.3. Statistical Analysis

To determine what contributes most to discrimination between groups with different intentions to vaccinate, we performed discriminant analysis on three groups of participants [51]. One group were those who were already vaccinated (fully vaccinated, *n* = 250), the second group was composed of those who were not yet vaccinated and were unsure whether they would become vaccinated or not (vaccine-hesitant, *n* = 99), and the third were those who were not vaccinated and said they had no intention to become vaccinated (vaccine-refusing, *n* = 132). With the discriminant analysis, we tested whether demographic variables and COVID-19-specific phenomena (COVID-19-related anxiety and conspiratorial thinking regarding COVID-19) can contribute to the differentiation of these three groups. Categorical variables were included as the dummy variables (gender: male vs. female; political orientation: center vs. left and center vs. right); for the ordinal variables, polynomial coding was used [52], i.e., the linear and quadratic trends were tested for education, importance of religion, financial status, and size of place of residence, while the other variables were treated as continuous discriminating variables (age and COVID-19-specific phenomena). In order to test the replicability of the discrimination accuracy [51], the discriminant analysis was performed on a randomly chosen 60% of the original sample (the training set, *n* = 296). The rest of the sample (the test set, *n* = 185) was then used to test the accuracy of the classification based on the discriminant model established in the first part of the sample. The analyses were performed with jamovi 2.2.5.0 [53].

## 3. Results

### 3.1. Participant Characteristics

The participants were on average median = 23 years old (*IQR* = 4, range 18–69, skewness = 2.80 (*SE* = 0.11), kurtosis = 8.62 (*SE* = 0.22)). Most of this sample were women and students (Table 1). They were of various attained education levels, about half of the sample lived in larger cities, and the participants mostly considered their income to be average (Table 1). The participants showed medium levels of COVID-19-related anxiety (median = 3, *IQR* = 1.2, skewness = −0.30 (*SE* = 0.11), kurtosis = −0.20 (*SE* = 0.22)) and low levels of conspiratorial thinking regarding COVID-19 (median = 2.17, *IQR* = 1.83, skewness = 0.44 (*SE* = 0.11), kurtosis = −0.81 (*SE* = 0.22)).

### 3.2. Discriminant Analysis

The discriminant analysis indicated that the three groups could be discerned by two dimensions, of which the first discriminant function explained 89.2% of the group differences, and the second function only 10.8%. Therefore, we concluded that the first function is the one most relevant for the discrimination between these groups. This function was mostly defined by conspiratorial thinking regarding the COVID-19 scale, although political orientation, financial status, and the COVID-19-related anxiety scale also showed a certain rate of discriminative power (Table 2). Vaccine-refusing participants had lower results on this function, those who were fully vaccinated had higher results, and vaccine-hesitant participants mostly varied around the average. Therefore, this function implied that one of the larger differences was that the vaccine-refusing group tended to have higher levels of conspiratorial thinking regarding COVID-19, but also that they were less prone to left political orientation, had a lower financial status, and lower COVID-19-related anxiety. 

When predicting group membership based on these functions, the prediction accuracy was higher than the 33.3% expected by chance for the vaccine-refusing and the fully vaccinated group (Table 3), but it was quite low for the vaccine-hesitant group. This indicates that the used variables do not contribute to the differentiation of vaccine-hesitant participants from the other two groups, although they can explain some of the differences between the vaccine-refusing and the fully vaccinated group. The accuracy of the discrimination was tested by using the same function to discriminate between these three groups. However, in the test set (*n* = 185), the prediction accuracy was a bit lower, as is to be expected due to sample bias [51], but not by much, indicating that these variables contribute to the differentiation of these groups independently of the sample used.

## 4. Discussion

The main finding of this study was the fact that conspiratorial thinking regarding COVID-19 contributed by far the most to the discriminant function explaining the differences between vaccinated individuals and those refusing vaccination. This finding is in line with the one we obtained in a similar population of young adults but a year earlier, before the vaccine against SARS-CoV-2 became widely available; among the sociodemographic predictors and the ones pertaining to the perceived vulnerability to the disease, the only significant predictor of the intention to receive the vaccine once it becomes available was the trust in science [22]. This is also in accordance with a systematic literature review by Ripp and Röer [44]. Since the tendency for conspiratorial thinking and the lack of trust in science and scientists are highly interrelated concepts [16], these two findings reflect the same mentality and highlight the importance of interventions aiming at rebuilding the trust between public and health/scientific authorities. As described in the introduction, COVID-19-related conspiracy theories are abundant and range from less credible ones, such as the pandemic being a made-up hoax, to more easily endorsed ones, such as the pandemic-related crisis being exaggerated for political reasons. From a laymen viewpoint, there were numerous scientific inconsistencies in the messages the health authorities sent since the beginning of the COVID-19 pandemic. This fueled mistrust in scientists and science, as can be seen from the fact that COVID-19-related conspiracy theories are often centered around the dismissal of scientific research (e.g., [7]). Higher enthusiasm about science was shown to predict more knowledge and less misleading reasoning regarding COVID-19, whereas science skepticism is related to more false beliefs about the pandemic and less support for a biomedical approach [8]. Van Mulukom et al. [9] suggested that the belief in COVID-19 conspiracy theories may be boosted by the low levels of trust in science in a context of threat and low levels of extensive and obtainable information in the unprecedented context of insecurity.

Recently, based on the notion that factual arguments can be effective in reducing conspiracy beliefs only in its early stages and before a conspiracy theory has taken its root (e.g., [54,55]), a number of so-called psychological inoculation techniques have been proposed. These consist of simple interventions such as warnings about fake news and pre-exposure to weakened doses of the techniques used in the production of fake news and have been shown to be effective in boosting psychological resistance against the endorsement of pseudoscientific narratives (see [55,56,57]. In planning future vaccination campaigns, policy makers might opt to act pre-emptively and employ some of these inexpensive techniques in order to boost psychological resistance against the endorsement of detrimental conspiracy theories and/or pseudoscientific facts regarding vaccination. 

Apart from the conspiratorial thinking, the next highest discriminatory power in this study was observed for the political orientation, with the vaccine-refusing group leaning less to the left. Previously, it has been shown that conspiracy beliefs are found on both sides of the ideological spectrum, and the general belief in conspiracy theories is strongest at either extreme of the political spectrum [58]. Regarding COVID-19, conservatism was found to be associated with lower perceptions of vulnerability to the virus and a stronger belief that the impact of the virus is exaggerated by the media. Furthermore, conservatives showed lower and less accurate knowledge of COVID-19 and were poor in disentangling real from fake news, e.g., [5]. 

Surprisingly, health-related anxiety showed substantially lower levels of discriminative power than conspiratorial thinking regarding COVID-19 (Table 2). Since the intent to vaccinate depends upon people’s perception of their risk to contracting a disease [59], we expected this variable to show a discriminative power similar to the one obtained by the conspiratorial thinking, albeit in the other direction (i.e., making the decision to become vaccinated more likely). Marinthe et al. [23] showed that the link between a conspiratorial mentality and the perception of risk may not be a straightforward one, as the perceived risk of death and the motivation to protect oneself can act as a suppressor of conspiratorial thinking, thus resulting in normative compliance after all. The vaccine-refusing group also had a somewhat lower educational status and placed higher value on religion; however, these two variables showed a modest discriminative power. 

Another potentially important insight from this study is the fact that the members of the vaccine-hesitant and vaccine-refusing group were quite dissimilar, reflected in the fact that the vaccine-refusing group had a very high percentage of correct classifications based on their discriminant scores, while the prediction accuracy for the vaccine-hesitant group was not higher than the one expected by chance (Table 3). This implies that targeted interventions should take into account whether their aimed recipients already hold some firm beliefs regarding vaccination (the vaccine-refusing group) or are unsure and baffled by the multitude of various sources, whose legitimacy they are not able to assert (the vaccine-hesitant group). For those who are vaccine-hesitant, it would be helpful to establish the phase of vaccine hesitancy and adjust the interventions accordingly [42]. Building on previous experiences on the development of adult vaccination programs through time and having a governmental working group specialized for adult vaccination would also be beneficial, seeing how adult vaccination is usually less endorsed than child vaccination, e.g., [60,61]. Interventions should focus specifically on disease prevention and the health security of adults [61], taking into account that individual and group influences, including the attitudes and beliefs related to vaccination, are the most frequently reported barriers to vaccination [62].

Furthermore, even though the accuracy of predicting group membership for the vaccine-refusing and fully vaccinated was satisfactory, there were still participants which were categorized in the wrong group. This indicates that there are other variables that contribute to their differentiation. These could include other psychological variables, such as general or existential anxiety [63], but also health status, due to which vaccination is not a viable option for a small part of the population [64], and societal context, i.e., the social media exposure [65]. It is also important to note that although the discriminant analysis we performed allowed for the testing of the linear and certain polynomial trends, it did not test for possible interaction effects. For example, Boon-Itt et al. [64] showed that the relation between age and vaccine intention varied with health status. 

*Limitations and the strengths of the current study*. As other online studies, this study failed to enroll an unbiased sample of participants: the ones who decided to participate were generally more educated, were mostly women, had a higher financial status, and did not harbor extreme political views. Additionally, since the data presented here were only a part of a larger study aiming to assess the pandemic behaviors among young people, due to the length of the questionnaire, the dropout rate was substantial, and thus the sample size on which these analyses were made makes the study somewhat underpowered. When interpreting the results of this study, one should also take into account the content of the conspiracy beliefs. While almost all COVID-19 conspiracy beliefs are related to vaccination willingness, the strength of these relations varies depending on the content of the belief, with, for example, the microchip implantation belief used in our research being a stronger predictor than the belief regarding SARS-CoV-2 being human-made [44]. Our research, however, contributes to the field which shows that conspiracy items have a strong one-factor structure, which is an aspect of validity rarely tested when exploring conspiracy beliefs [66]. This structure is in accordance with the previously established systematicity of the relationship of different conspiracy beliefs and vaccination willingness [44]. Furthermore, the participants’ experience with COVID-19 was limited; however, for the purpose of designing future interventions aimed at rising vaccine acceptance rates among the general, healthy population, this is not necessarily a shortcoming (as older, chronically ill, and vulnerable populations usually receive more one-to-one health care and information from their primary physicians). We believe that the timing of the data collection for this study represents another strong point for drawing conclusion regarding the vaccination decisions: at that point (May/June/July, 2021), the mass vaccination campaign in Croatia had only just gained momentum for the healthy and younger individuals (in the months preceding this point, only healthcare workers, nursing home residents, and chronically ill patients were eligible for the vaccine), and thus the insecurity regarding the decision and the perception that there is not enough information was higher than it would be after the number of fully vaccinated people around the globe rose significantly later on. 

## 5. Conclusions

In conclusion, our study showed that the conspiratorial thinking regarding COVID-19 held the highest discriminative power in explaining the differences between vaccinated individuals and those refusing vaccination. Other variables, such as demographic characteristics and COVID-19 health anxiety, were by an order of magnitude (in terms of the coefficients of the discriminant function sizes) less informative. In planning future vaccination campaigns, policy makers might opt to act pre-emptively and aim to boost the psychological resistance against the endorsement of pseudoscientific narratives, for example, by employing techniques of psychological inoculation against fake news. Furthermore, the study showed that individuals who have already decided to either refuse or receive a vaccine against SARS-CoV-2 were easily distinguishable based on the variables used in this study, while the ones in the vaccine-hesitant (unsure) group probably had more heterogeneous reasons contributing to their indecisiveness and, as such, represent a group which future studies should aim to explore further. 

## Figures and Tables

**Table 1:** Sociodemographic characteristics of the participants (*N* = 481).

Characteristic	Category	%
Gender	Male	18.1%
	Female	81.9%
Employment status	Employed	27.9%
	Unemployed or retired	5.0%
	Students or pupils	67.2%
Education attainment	Primary or high school	37.6%
	College or bachelor	40.7%
	Master or doctoral	21.6%
Size of place of residence	<10,000	23.7%
	10–100,000	28.3%
	>100,000	48.0%
Household income	Below average	10.4%
	Average	67.4%
	Above average	22.2%
Importance of religion	Low	52.6%
	Mid	15.6%
	High	31.8%
Political orientation	Left	44.1%
	Center	43.2%
	Right	12.7%
Intention to vaccinate	Fully vaccinated	52.0%
	Vaccine-hesitant	20.6%
	Vaccine-refusing	27.4%

**Table 2:** Coefficients of the first discriminant function.

Discriminant Variable	Coefficient
Conspiratorial thinking regarding COVID-19	0.852
Left political orientation	0.486
Financial status (linear relationship)	0.435
COVID-19-related anxiety	0.423
Education (quadratic relationship)	0.304
Importance of religion (linear relationship)	0.301
Financial status (quadratic relationship)	−0.234
Education (linear relationship)	−0.215
Place of residence (linear relationship)	0.191
Female gender	−0.178
Importance of religion (quadratic relationship)	−0.149
Place of residence (quadratic relationship)	0.142
Right political orientation	−0.054
Age	−0.020

**Table 3:** Accuracy of the discrimination in the training and the test set.

Group	Predicted Group Membership			Total	Percentage Correctly Classified
	Vaccine-Refusing	Vaccine-Hesitant	Fully Vaccinated		
Vaccine-refusing	61/33	5/10	17/9	83/52	73.5/63.5%
Vaccine-hesitant	9/5	16/3	37/8	62/16	25.8/18.8%
Fully vaccinated	18/11	6/24	127/82	151/117	84.1/70.1%

Note. The frequencies are reported for the training/test set.

## Data Availability

The data presented in this study are available on request from the corresponding author.
